# The predictive factors of US hospital bankruptcy - a multi-model comparison

**DOI:** 10.1007/s10729-025-09750-6

**Published:** 2026-02-21

**Authors:** Brad Beauvais, Zo Ramamonjiarivelo, C. Scott Kruse, Lawrence Fulton, Ramalingam Shanmugam, Arvind Sharma, Aleksandar Tomic

**Affiliations:** 1 Texas State University, School of Health Administration, Encino Hall, Room 250 A, 601 University Drive, San Marcos, TX 78666 USA; 2 College of Health Sciences, University of Texas El Paso, 500 W. University Ave, El Paso, TX 79968 USA; 3 Boston College, Woods College of Advancing Studies, St. Mary’s Hall South, 140 Commonwealth Ave., Chestnut Hill, MA 02467 USA

**Keywords:** Hospital bankruptcy, Financial insolvency, Debt, Predictive model

## Abstract

In response to the growing number of hospital bankruptcies across the United States, this study sought to develop a predictive and interpretable model tailored specifically to the healthcare industry. Utilizing a longitudinal dataset of 3,091 short-term acute care hospitals from 2008 to 2021, we evaluated and compared traditional bankruptcy prediction models—Altman's Z'', Ohlson’s O-score, and Zmijewski’s model—against a newly developed hospital-specific logistic regression model (BRKFSST). We incorporated over 30 financial and hospital-level variables, including quality indicators, ownership type, and market characteristics. Unlike prior models, ours lagged all unknowable variables to ensure true out-of-sample prediction. The BRKFSST model achieved strong performance, with an Area Under the Curve (AUC) of 81.8%, balanced accuracy of 72.2%, and a mean recall of 60.6% across multiple test/train splits, outperforming all benchmark models. Importantly, the model retained interpretability, allowing for the identification of key predictors such as labor compensation ratio, adjusted patient days, and quality ratings. These findings provide actionable insights for hospital leaders and policymakers to identify at-risk institutions and implement early interventions to prevent financial collapse and preserve access to care.

## Introduction

In the United States, short-term acute care is delivered by a wide variety of hospitals and health systems. Unlike other developed nations, the US has no single central planning or controlling organization for hospitals. Instead, hospitals in the US ensure long-term economic viability by providing as many visits and services as possible in a predominantly fee-for-service system. As with any business, this revenue model is structured to generate adequate cash flow to cover operational costs. However, in recent years, many hospitals across the country have not been able to maintain profitability, and bankruptcy has become increasingly common [1]. The reasons behind this trend are varied, originating from numerous macroeconomic, demographic, and policy factors. Among other causes, declining government-sponsored insurance reimbursement, rising staffing costs, increased technology costs, heightened demand from the uninsured, and intensified competition have imposed heightened pressure on hospitals across the country [2].

During the COVID-19 pandemic, healthcare facilities were challenged further by the loss of revenue from more profitable diagnostic, outpatient, and ancillary services. This additional financial strain, coupled with drastically higher operating costs, crippled many hospitals. The Polsinelli quarterly report pertaining to financial distress noted that although bankruptcy filings in most industries have decreased by 53% nationwide since 2010, filings in the healthcare industry have increased by 305%, with a disproportionate number associated with hospitals in rural settings [1]. The Chartis Group also recently reported that nearly half of rural hospitals operated at a loss and that 138 rural hospitals have closed since 2010, even before the effects of the COVID-19 pandemic were financially realized [2]. Similar concerns of hospital bankruptcy exist in urban settings, particularly regarding safety net hospitals, which predominantly serve the nation’s urban poor. Like their rural counterparts, these facilities have also struggled to remain viable. Without the requisite financial reserves to support operations, bankruptcy and closure have become the only viable options.

Bankruptcy of any type can create negative effects on the organization and the community. First, it irrevocably damages the organization’s financial credibility and makes it very challenging for the organization to obtain creditors’ confidence in the future. Second, bankruptcy may lead to staff downsizing, restructuring, and layoffs which can negatively affect the economy at the local, regional, and national level, depending on the size of the organization. Third, if the organization ultimately closes, the effects are even more profound. The loss of a hospital drastically reduces access to healthcare services and may also result in neighboring hospitals having issues accommodating the influx of former patients from the bankrupt/closed hospital. Additionally, many government-owned public hospitals are safety net providers. Thus, the immediate consequence of closure is diminished access to care for the underinsured and uninsured population. And if a public hospital is the sole community provider, the community is left with no access to hospital care, and consequently, public health can significantly deteriorate [3].

While hospital bankruptcy can result in a myriad of problems for patients, providers, and the local economy, it is a preventable situation if the threats causing financial distress can be accurately detected and appropriately addressed before bankruptcy becomes the only managerial option. The purpose of this study is to evaluate and build upon prior bankruptcy studies by developing an explanatory and predictive model of hospital bankruptcy using a robust longitudinal sample of hospitals drawn from across the United States.

This study makes several important contributions. First, it develops a hospital-specific predictive bankruptcy model (BRKFSST) utilizing a robust dataset that incorporates both financial indicators and critical hospital-specific variables, such as quality ratings and market characteristics. Second, it demonstrates superior predictive accuracy compared to traditional bankruptcy prediction models like Altman’s Z'', Ohlson’s O-score, and Zmijewski’s model. Finally, it provides actionable, interpretable insights to assist hospital leaders and policymakers in proactively managing financial risk.

## Literature review

### Bankruptcy concepts, prediction models, and methodological evolution

The term ‘bankruptcy’ is based on the legal proceeding that commences when an individual or company is incapable of repaying its debts or financial obligations. The type of bankruptcy proceeding an organization opts to pursue depends on the firm’s situation and legal structure. There are three types of bankruptcy: ‘Chapter 7’, ‘Chapter 11’, and ‘Chapter 13’. Each form of bankruptcy is drawn from different “chapters” in the U.S. Bankruptcy Code. Under ‘Chapter 13’ of the bankruptcy code, sole proprietorships can reorganize assets and liabilities. This allows the business owner to repay creditors within three to five years. In contrast, partnerships and corporations can file for bankruptcy protection under ‘Chapter 7’, which ceases operations of the business, or ‘Chapter 11’, which is a reorganization and allows the business to continue to operate [4].

The factors that can force an organization into bankruptcy have been of interest for nearly a century. Bellovary et al. [5] reviewed the slate of bankruptcy prediction studies from 1930 and found a total of 165 different models to consider. Prior researchers have developed various methods to attempt to forecast a firm’s likelihood of going bankrupt [3, 6–8]. Financial ratios were used to predict bankruptcy, including capital/total assets, cash/total assets, reserves/total assets, sales/total assets, current assets/total assets, the current ratio, fixed assets/total assets, net profit/total assets, net worth/fixed assets, net worth/total assets, and net worth/total debts.

Beaver suggested the ratio of cash flow over total debt, which measures the relationship between a company's operational cash flow and its total debt, as the optimal predictor of organizational bankruptcy. Altman [9] employed discriminant multivariate analysis to evaluate and develop a set of single equations as models to predict organizational bankruptcy in different industries such as manufacturing and service industries. From his analysis, Altman derived a financial index, the Z-score model, which can be used to forecast the likelihood of a company going bankrupt within the next two years. The original Z-score model is Eq. (1). It is comprised of the combination of five weighted ratios from five areas of organizational financial performance: profitability, leverage, liquidity, solvency, and activity, with a composite Z-score of 1.80 or below indicating a higher likelihood of bankruptcy.

1 $$\text{Z }= 0.012\times \mathrm{X}1 + 0.014\times \mathrm{X}2 + 0.033\times \mathrm{X}3 + 0.006\times \mathrm{X}4 + 0.999\times \mathrm{X}5$$

where X1 = working capital/total assets, X2 = retained earnings/total assets, X3 = earnings before interest and taxes/total assets, X4 = market value of equity/total liabilities, and X5 = net sales/total assets.

Altman expanded his set of models for use with smaller private manufacturing companies and non-manufacturing companies with assets less than $1 million. He titled these models the Z′-score and the Z″-score. The Z′-score was developed specifically for private manufacturing companies, while the Z″-score was created for non-manufacturing firms, which might include organizations in the service sector such as hospitals and other healthcare organizations. This revised model is Eq. (2).

2 $${\mathrm{Z}}^{{\prime}{\prime}} = 6.72 \times \text{ X}1 + 1.05 \times \text{ X}2 + 6.5 \times \text{ X}3 + 3.26 \times \text{ X}4$$

where X1 = working capital/total assets, X2 = retained earnings/total assets, X3 = earnings before interest and taxes/total assets, and X4 = market value of equity/total liabilities.

From a critical standpoint, Altman’s models were built on artificially balanced data (i.e., the same number of bankruptcy and non-bankruptcy cases), thus any performance metric (e.g. accuracy) calculated on this balanced data is biased. Altman also estimated models using the entirety of the collected data and under-explicated assumptions (e.g., Gaussian distribution of the data supporting discriminant analysis). This is an assumption unlikely to hold as noted by Ohlson [10]. By using the entirety of the data, the model is explanatory rather than predictive. Further, Altman used information from time *t* to forecast information at time *t* when building the model. The model was initially built as a post-mortem assessment rather than a priori predictor.

3 $$\begin{aligned}\text{O }&=-1.32-0.407\times \mathrm{O}1 + 6.03 \times \text{ O}2\\&-1.43 \times \text{ O}3 + 0.0757 \times \text{ O}4-2.37 \\& \times \text{ O}5-1.83 \times \text{ O}6 + 0.285 \times \text{ O}7\\&-1.72 \times \text{ O}8-0.521 \times \text{ O}9\end{aligned}$$

where O1 is the logarithm of TA divided by the gross national product (GNP) price-level index assuming a base value for 1968, O2 is the total liabilities (TL) divided by total assets (TA), O3 is working capital (WC) divided by TA, O4 is current liabilities (CL) divided by current assets (CA), O5 is an indicator variable if TL are greater than TA, O6 is net income divided by TA, O7 is operating funds divided by TL, O8 is an indicator variable for two consecutive years of negative net income, and O9 is the change in net income divided by the absolute values of net income at year t-1 and year t-2 [10].

Ohlson improved Altman’s work by offering a score that predicts bankruptcy using ratio and indicator variables that are actually lagged (Eq. 3) [10]. In estimating his model, Ohlson applied logistic regression (LR), and his work was an improvement over Altman. The author leveraged data prior to the organization declaring bankruptcy and used one vector of observations for a single firm-year selected via random sampling, although Ohlson correctly notes that all firms that met the inclusion criteria should have been used. Once again, all data were used rather than preserving a test set for evaluating accuracy metrics, and the assumptions of logistic regression were largely ignored (possibly for ease of interpretation). The use of logistic regression itself, however, was also an improvement over the Altman model.

While investigating empirical bias in financial distress models, Zmijewski provided an additional method to predict bankruptcy while investigating the proportion of non-bankrupt firms to be included in the training sample. In contrast to earlier models, Zmijewski employed probit models of 40 bankrupt and 800 non-bankrupt firms in a training set and 41 bankrupt and 800 non-bankrupt in a validation set [11]. Six ‘choice-based’ samples were drawn from the above training set, manipulating the number of non-bankrupt selections to the set {40, 100, 200, 600, 800} to investigate model accuracy metrics under various proportional sampling schemes. Unfortunately, Zmijewski did not use time-lagged variables, so prediction was assumed to be independent of the firm’s prior financial performance. Also, the author’s variables were notably just a subset of Ohlson’s variables. Nevertheless, Zmijewski’s contribution demonstrated that biased selection of non-bankrupt firm counts affects model accuracy metrics immensely, even when using no time lags. For example, using 800 non-bankrupt firms versus 40 non-bankrupt firms and non-lagged variables reduces the accuracy in ‘prediction’ (explanation) of bankruptcy from 97.6% to 70.7%. The model consists of the following variables:

4 $$\text{Zmijewski Score }= -4.336-4.513\times \mathrm{O}6 + 5.679\times \mathrm{O}2 + 0.004\times 1/\mathrm{O}4$$

where O6 = net income divided by TA, O2 = total liabilities divided by TA, and O4 = current liabilities (CL) divided by current assets (CA).

Zmijewski highlighted that other bankruptcy scoring models oversampled distressed firms which inflated their accuracy metric. Although his model includes only three ratios, it has been shown to perform better in some studies than the Altman model; however, it is a subset of the Ohlson model, with only one term being a reciprocal (Current Assets/Current Liabilities). The use of a reduced set of predictors does not necessarily inhibit accuracy or model performance. This is supported by the work of Bellovary, Giacomino and Akers [5] who observe that higher model accuracy is not assured with a greater number of factors. Some of the models they reviewed with two factors were just as capable of accurate prediction as models with 21 factors [5]. Unfortunately, the authors looked only at accuracy, not precision, recall, or other metrics, which are more meaningful for imbalanced data.

### Internal and external factors associated with US hospitals’ financial distress & bankruptcy filing

Historically, bankruptcy studies have focused on non-healthcare service organizations and manufacturing firms. Organizational financial distress and bankruptcy are equally as important in the healthcare sector. It could be argued that the limited number of studies of healthcare organizations is attributable to the complexity of the regulatory, reimbursement, and workforce challenges faced by hospitals across the country. Early research on the topic tended to focus on financial contributory factors. For example, McCue [12] studied California hospitals and concluded that profitability rather than cash flow was a better predictor of financial distress [12, 13]. In a descriptive, case-by-case study, Bazzoli & Cleverley [13] found that negative cash flow, low cash flow/total debt, low current ratios, negative equity, and loss of profitability were likely to negatively influence economic viability in the 11 hospitals studied [12, 13]. In a descriptive comparison of ‘financially distressed’ hospitals (those with BBB- Standard & Poor’s ratings which existed in the American Hospital Association Directory), Bazzoli and Andes (1995) found that these facilities had access to fewer assets, encountered negative profitability in terms of both total margin and return on assets, and sustained high debt loads and poor liquidity in comparison with more financially secure institutions [14]. These authors also indicated hospitals that filed for bankruptcy and permanently closed their doors (e.g., Chapter 7 bankruptcy) were more likely to be for-profit hospitals and have a lower cash flow/total debt ratio [14].

Some earlier researchers have also attempted to apply predictive models to assess the likelihood of bankruptcy of hospitals. These studies have included the Financial Strength Index [15], the Altman Z-score model [16–19] and the Ohlson O Score [10], and the Zmijewski score [11]. However, when Corbett and Gosset [18] assessed the effectiveness of financial and non-financial variables in predicting the financial solvency of private for-profit hospitals in the US and included predictive models including the Altman Z-score, they found that none of the tested models, nor the financial and non-financial ratios, were significantly associated with for-profit hospital financial solvency [18]. Puro et al. [19] further assessed the predictive accuracy of three models including the modified Altman Z-score and the Ohlson O score, but they also did not find a single ratio that consistently separates bankrupt and non-bankrupt hospitals across the tested models. Notably, none of these models include time-lagged variables for prediction, thus they are purely explanatory.

Most studies of bankruptcy have relied solely on financial data to derive their models. Given the lack of predictive capability in current models, we posit that there are other identifying features of hospitals that should be considered in the financial viability assessment and prediction process. In more recent research, hospital financial distress has been associated with low occupancy rates, slow collection of account receivables, poor payer mix in terms of a high percentage of Medicare and Medicaid patients, aging facilities, poor management, fraud allegations, demographic changes, financial strategy/desire to sell, quality issues, physician malpractice insurance, physician politics, and external politics [20–23]. Market and environmental factors have also been shown to be significantly associated with government-owned/public hospitals’ financial distress. These factors include increased Medicare Advantage penetration at the county level, increased competition, workforce issues, demographic changes, economic conditions, and external politics [24, 25]. Lastly, quality has not been widely used in former hospital bankruptcy studies; however, there is good reason to believe quality of care plays a factor in determining hospitals’ financial status, especially in the current value-based care context. Thus, including measures such as the Center for Medicare & Medicaid Services Overall Hospital Quality Star Rating as one of the predictor variables is a logical step. The star rating is a composite score that reflects a hospital's performance across several safety, efficiency, patient experience and effectiveness metrics which may be linked to financial performance [26–28].

Based on our literature review, it is apparent that existing models lack predictive capability as they apply to the hospital setting. Further, of the tested models, only a few incorporate a robust set of external and internal market and organizational characteristics. For these reasons, we seek to build on our knowledge of prior authors’ discoveries pertaining to bankruptcy, and hospital bankruptcy, in particular. In our efforts, we seek to identify which independent variables among existing bankruptcy prediction models (i.e., the Altman Z-score model for non-manufacturing firms, the Ohlson’s O model and the Zmijewski’s Z model) remain salient within the hospital context as the unit of analysis. We also intend, for prediction purposes, to include all unknowable random variables as lagged variables in order to be predictive rather than explanatory. Given the difficulties previously mentioned in prior authors’ research efforts, we also intend to expand the set of analysis variables to identify which internal and external factors are relevant to hospital bankruptcy studies. Through this analysis we seek to establish a foundation from which future research might be developed to construct explanatory and predictive models for hospital bankruptcy. With the important role hospitals serve in society, and recent changes in health policy and reimbursement in the United States, we consider it an important endeavor to clarify what might cause these organizations to succumb to financial pressures. This knowledge may help healthcare policy makers, hospital leaders, creditors, investors, and the public more clearly understand which hospitals are at risk and plan accordingly.

## Methods

### Data, unit of observation, & sample size

Data were acquired by custom query from Definitive Healthcare for the years 2006 through 2021 [26]. These data were joined by hospital identifier with the Centers for Medicare and Medicaid (CMS) Hospital Compare data [27]. Most of the financial data are available through CMS Hospital Provider Cost Reports [28], although Definitive uses a parochial estimation model for facilities not reporting to CMS. The initial sample size was 47,136 hospital-year observations of short-term acute care hospitals in the U.S. All Federal hospitals, including 172 Veterans Affairs, 26 Indian Health Service, and 31 Military Health System facilities, were excluded from our study sample due to a lack of numerous relevant data elements. After removing observations with missing columns and rows of 25% or more, the final sample size was 46,855 hospital-year observations, which equated to 3,196 hospitals. Of these hospitals, 71 had declared bankruptcy one or more times during the period (only the first bankruptcy was used). Thirty-four hospitals did not have observations for all years due to closure, bankruptcy, opening, or other factors. After eliminating those facilities with greater than 20% missing columnar data, hospitals with fewer than three years of consecutive observations, and facilities located in U.S. territories, the set was reduced to 3,091 hospitals with 65 unique bankruptcies.

Our study leverages short-term panel data, where the predictive framework inherently depends on the temporal nature of the dataset. Following methodologies like Ohlson [12] and guidance from Dormann and Griffin [29] on optimal time lags, we focus on recent temporal observations to ensure robust predictions. The dataset spans 2006–2021 but is inherently ragged, as facilities with limited observations, particularly those bankrupt early in the period, could not contribute meaningfully to the analysis. By restricting the window to a three-year panel, we retained 65 facilities with sufficient data, minimizing bias associated with disproportionate exclusion of facilities with shorter histories. This design also aligns with decay of influence (e.g., exponential decay) where older observations have diminished predictive value. Furthermore, machine learning techniques employed, such as Support Vector Machine (SVM) and Perceptron, necessitate non-ragged data for matrix operations, making this approach both practical and methodologically sound. While we acknowledge the limitation of excluding longer temporal horizons, alternative methods, such as zero-inflated, left- and right-truncated survival analysis, as well as dynamic panel analysis, offer potential for future research to explore richer temporal dynamics. Our study prioritizes analytical rigor while balancing data constraints to advance predictive modeling in hospital management research.

Vital to note in this study is that unlike many studies, all unknowable, random predictors were lagged. No unknowable information about bankruptcy at year *y* was used to predict*.* Only information from year *y-1* and *y-2* were used to predict bankruptcy for year *y*. This statement holds true for the model comparisons that follow (e.g., Altman). For example, Altman did not use lagged variables in the initial model estimation; however, we used lagged variables to evaluate fairly the Z’’ predictive capacity (both with the original coefficients and re-estimated coefficients). In doing so, the model truly investigates ex-ante predictive capability, not posterior explanatory information.

For these 3,091 hospitals, three consecutive years of observations were included. For non-bankrupt hospitals, the starting year of observations was selected via random sampling. Then the two previous years of information were gathered to be used as potential predictors. Thus, the entire population of hospitals given the inclusion criteria were represented in the analysis. For those facilities entering bankruptcy in year *y*, data from years *y-1* and *y-2* served as potential predictors. Thus, bankruptcy status at year *y* was modeled as a function of data gathered from year *y-1* and year *y-2*.

### Software

Python 3.X [30] and R [31] served as the analytical tools for both explanatory and predictive models. R Studio (Posit) provided the integrated development environment. The individual packages and libraries used are cited online where available.

### Variables - dependent variable (DV)

The dependent variable (DV) was bankrupt status, a Bernoulli trial. The coding for the DV is as expected: {0 = not bankrupt, 1 = bankrupt}.

### Variables - independent variables (IVs) – traditional models

One set of predictors was Altman’s predictors of bankruptcy for non-manufacturing firms (Eq. 1). A second set of predictors were the Ohlson’s O-score independent variables (Eq. 2). And a third set included Zmijewski’s Z Variables (Eq. 3). For their original models, we evaluated lags at *y-1* only to provide true ex-ante predictions.

A final set of predictors was chosen for an alternative, hospital-specific model. The variables in Table 1 plus numerous independent variables were included in the study to account for the variation in hospital bankruptcy associated with various individual hospital and hospital market characteristics, including the number of hospital discharges, adjusted patient days, number of staffed beds, hospital ownership type (for profit vs. not-for-profit), government-operated or not, average daily census, average length of stay, local hospital market concentration (as measured via the Herfindahl–Hirschman Index), the hospital case mix index, the medical case mix index, the surgical case mix index, the Complications or Comorbidities (CC) and Major Complications or Comorbidities (MCC) rate, the bed utilization rate, the labor compensation ratio, urban or rural location, Medicare percent payer mix, Medicaid percent payer mix, the hospital serious complication rate, the Hospital Value Based Purchasing Total Performance Score, and the Hospital Consumer Assessment of Healthcare Providers and Systems Star Rating. The variable selection procedure is forthcoming.

**Table 1:**
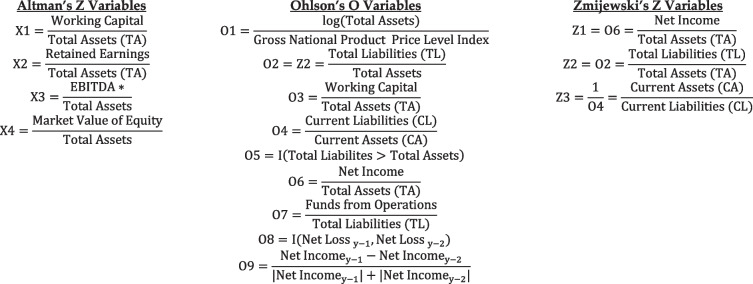
Original predictors for Altman, Ohlson, and Zmijewski

*EBITDA earnings before interest, tax, depreciation and amortization

## Models

**Fig. 1:**
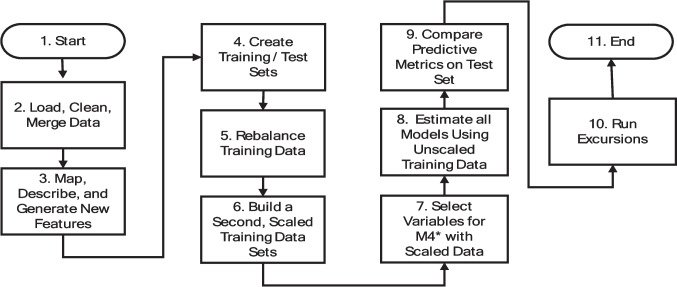
Process diagram for the analytical flowchart

The process diagram for the entirety of the analysis is shown in Fig. 1 below.

In Fig. 1, pre-cleaned and transformed data were loaded and merged with the census data for geographical plotting. The number of hospitals included in the study was 3,091. Descriptives and correlations were generated, and features were engineered (e.g., dichotomous quality indicator). Noticeably missing is the investigation of transformations. We investigated power transformations (e.g., Box-Cox) along with many other engineering efforts; however, our intent was to keep the models as interpretable as possible. Thus, we avoided the infinite number of transformations available outside of discretization and scaling in favor of interpretability.

Part of the process involved the building of training and test sets. To evaluate the predictive capability of the models, the hospitals were initially split into a 50% training set and a 50% test set using a pseudo-random number seed and stratification based on bankruptcy status. Since the data were highly imbalanced, stratification ensured that a (nearly) equal number of bankrupt observations were in both groups.

The initial split was chosen because of the small number of bankruptcies available in the data (65 total) and the need to rebalance the training set. K-fold cross-validation (which *was* used on the training sets for alternative model variable selection) was not used on the entirety of the data as it required stratification and rebalancing for all combinations of training folds [32]. The rebalancing is necessary for models that are relatively inflexible (e.g., logistic regression) to be able to learn about the minority class. The study also investigates additional splits of the data, 60%−40%, 70%−30%, 80%−20%, and 90%−10%. Performance metrics and, where appropriate, coefficients were compared across splits. The training and test set sizes are shown in Table 2.

**Table 2:** Counts of observations for investigating training/test splits

Split %	Training bankrupt	Training not bankrupt	Test bankrupt	Test not bankrupt	Total
50–50%	32	1513	33	1513	3091
60–40%	39	1815	26	1211	3091
70–30%	45	2118	20	908	3091
80–20%	52	2420	13	606	3091
90–10%	58	2723	7	303	3091

### Balancing of the training set only

Only 65 of the 3,091 hospitals in the dataset (2.1%) experienced bankruptcy. To account for this issue, the majority weighted minority oversampling technique (*mwmote*) from the *imbalance* library in R was used on each of the partitioned training sets [32]. The *mwmote* algorithm is a technique that addresses hard-to-learn minority classes (e.g., noisy data) by identifying them, assigning importance weights to them based on their Euclidean distance from the majority class in feature space, and then generating synthetic observations similar such that samples lie within some minority class cluster (e.g., they are representative of the minority [32].)

Specifically, the algorithm uses k-nearest neighbors (*knn*) three times. The first *knn* (*kNoisy*) is run for each minority observation to find its k nearest neighbors (default is 5) from both majority and minority observations. The proportion of those observations that are majority (the ‘hardness’ score) is calculated. Then the top $\gamma$ percent of the hardness scores are used to select minority observations that are difficult to learn. [32]

Next, a second *knn* (*kMinority*) is then run for each minority observation to find the k nearest *minority* observations only. Distance weights (e.g., Euclidean) for the k minority observations and the initial observation are calculated, and these weights then are normalized (scaled to sum to 1) and used to generate new observations by interpolation between the original minority observation and its nearest neighbors. [32]

Finally, a third *KNN* (*k-Majority*) is used on the synthetically generated observations. If the ‘hardness score’ here is high, then the synthetic observation is moved in the direction of the original minority observation by a small step size. [32]

Although the training sets were balanced so that there were an equal number of bankrupt and non-bankrupt observations, the test sets were left imbalanced and unadulterated to estimate out-of-sample predictive capability and stability across split percentages. In this way, the predictive capability of the models on unseen data could be evaluated.

### Model building

Variable selection for model building was necessary to build parsimonious models that retained predictive capability. The universe of available fixed and random variables included time-related variables, geographical variables (and engineered variables), financial variables, quality variables, hospital characteristic variables, and others. A detail of those variables is forthcoming.

For the financial variables, the team chose to use past ratio data (two lags and the change in those two lags). This method improves upon zero (Altman) and one-lag (Ohlson and Zmijewski) models by investigating additional time-based effects (the second lag and the change in lags). The reason for using two lags is multi-fold. First, no hospitals were reported bankrupt in 2008 and 2009, the first two years of observations. Thus, selecting two lags allowed for complete dataset representation of the entirety of the data. Had three or more lags been selected, the third and subsequent lags would have to be estimated, confounding estimates. Also, some hospitals did not have long lags available (e.g., relatively new or re-branded). As is the case with most panel data, the most recent previous observations are likely to be the most relevant as well [29]. Thus, to improve upon existing methods, to retain a complete dataset without missing lags, and to provide the most relevant predictions, the study uses two lags. Equation 5 depicts the equation we estimated to both explain and predict bankruptcy [29].

We used 3-fold, stratified cross-validation and ElasticNet regularization for a general linear model (logistic regression) to select variables. We produced separate estimates for each training split. ElasticNet (or ENet) extends the typical logistic regression estimation by adding two penalty functions to the estimation and is a useful tool for variable selection [33, 34].

5 $$min\\ \beta \left\{\begin{array}{l}-\frac{1}{n}\sum_{i=1}^{n}\left({y}_{i}\mathrm{log}\left(\widehat{{p}_{i}}\right)+\left(1-{y}_{i}\right)\mathrm{log}(1-\widehat{{p}_{i}})\right)\\+ \lambda \left((1-\alpha )\sum_{j=1}^{p}{\beta }_{j}^{2}+\alpha \sum_{j=1}^{p}\left|{\beta }_{j}\right|\right)\end{array}\right\}$$

In Eq. 5, the first term in parentheses is the traditional log-likelihood estimation problem for logistic regression. The second term is the Lagrangian penalty function ($\lambda$) for the mixed combination (parameter $\alpha$) of the L2 Norm (squared coefficients used in ridge regression) and L1 Norm (absolute value of the coefficients used in Lasso regression). Since the objective function seeks to minimize, some coefficients are driven to zero given the diamond shape (in high dimension) Lasso penalty. The ridge penalty (an ellipse in high dimension) is also included because it tends to handle correlated predictors better.

For each of the splits (50% through 90% training sets, see Table 2), coefficients were evaluated based on the results of the ElasticNet. The top 15 coefficients, based on absolute magnitude, were evaluated for inclusion. For highly collinear variables (e.g., correlation of 0.9 or higher), only one was selected for inclusion in the model. Variable selection for the model across all of the investigated splits was highly congruent as indicated by the ‘model votes’ column which counts the number of inclusions across the training splits. These variables were then selected for use in the alternative model based. Table 3 shows the congruence among models and model splits, with only the 50%−50% split model demonstrating any different variable selection choices.

**Table 3:** Elastic net congruence and variable selection

Variable	Model votes	Selected? 0 = No, 1 = Yes	Why
Adjusted Patient Days (y-1)	4	0	Collinear with Adjusted Patient Days at y-2 (r = 0.979)
Adjusted Patient Days (y-2)	4	1	
Altman X2 (y-2)	4	1	
AR/Op Income (y-1)	5	1	
AR/Op Income (y-2)	4	1	Not collinear
Bed Utilization (y-1)	5	0	Collinear with Bed Utilization at y-2 (r = 0.958)
Bed Utilization (y-2)	5	1	
Change in Liab/Fund Balance Ratio	4	1	
Change in Ohlson O4	5	1	
Days Cash-on-Hand (y-1)	5	0	Collinear with Days Cash-on-Hand at y-2 (r = 0.900)
Days Cash-on-Hand (y-2)	5	1	
Government status	5	1	
4 or 5 Star Facility (y-1)	5	1	
Labor Compensation Ratio (y-1)	5	1	
Ohlson O2 (y-2)	4	1	

### Model estimation – logistic regression

Logistic regression (LR) models served as the most appropriate tool for estimating variable directionality and magnitude. LR is well-suited for 2-class classification (e.g., bankrupt versus non-bankrupt) given the Bernoulli nature of each trial. In LR, the error term follows a logistic distribution. Using this distribution provides the following formula for estimating a bankruptcy: $P\left({Y}_{i}=1\right)= \frac{\mathit{exp}({\boldsymbol{X}}\beta )}{1+\mathit{exp}({\boldsymbol{X}}\beta )}$. Its complement is therefore $P\left({Y}_{i}=0\right)= \frac{1}{1+\mathit{exp}({\boldsymbol{X}}\beta )}$. The odds ratio (OR) is then $\frac{P\left({Y}_{i}=1\right)}{P\left({Y}_{i}=0\right)}$ which simplifies to $OR=\mathit{exp}({\boldsymbol{X}}\beta )$. By taking the log of the OR, the equation becomes linear in parameters,$log(OR)={\boldsymbol{X}}\beta$, and can be estimated with maximum likelihood estimation. LR avoids certain assumptions (e.g., homoskedasticity) but relies on linearity of log odds for continuous variables, absence of collinearity and extreme outliers, and independence of observations. These assumptions were investigated for the explanatory purposes of the study but were less important for the predictive component.

Other classifiers outside of logistic regression were also considered. Many of these models cannot provide coefficient directionality (e.g., typical tree/forest models) or are ‘black box’ (e.g., multi-layer, multi-node neural networks). Other models have necessary assumptions that are often difficult to meet such as multivariate normality of the predictors (e.g., multiple discriminant analysis, an assumption possibly violated in Altman’s research). The utility of logistic regression for this type of analysis is that it supports interpretability with few assumptions and without the requirement for scaling or transformations so long as assumptions are reasonably met. Still, we investigated a few other methods for estimating coefficients solely for our final model including a Perceptron (modified activation and loss functions), a support vector machine (SVM), and a tree model we call Unique Variable Decision Tree (SUVDT). The first two models do require variable standardization (e.g., min–max scaling), while the latter does not and involves manual building of trees where variables are selected for a split a maximum of one time per left or right branch, resulting in an interpretable decision tool. Appendix 2 contains descriptions and analysis of other methods explored.

### Metrics

All models were built on the augmented training set. These models were then used to forecast the test set. Typical classification metrics including balanced accuracy, precision, recall, specificity, F1-score, Area Under the Curve (AUC), and Precision-Recall Area Under the Curve (PR-AUC) were used to compare models along with side-by side confusion matrix plots. Table 4 details how the accuracy metrics were calculated.

**Table 4:** Definitions of the metrics used

Metric	Probabilistic/expectation definition
Balanced Accuracy	(Precision + Recall)/2
Precision (Positive Predictive Value)	Pr(+| Predicted +)
Recall (Sensitivity)	Pr(Predicted +| +), True Positive Rate
F1 Score	HM(Precision and Recall)
Specificity	Pr(Predicted—| -), Complement is False Positive Rate
Negative Predictive Value (NPV)	Pr(—| Predicted -)
Area Under the Curve (AUC)	Integral of AUC formed by Recall & 1-Specificity
Precision-Recall AUC	AUC for Precision versus Recall Curve
Misclassification Rate	Pr(Predicted +| -) + Pr(Predicted—| +)

*Note: "PR"* = probability, "+" = positive bankruptcy, "-" = negative bankruptcy, "*HM* harmonic mean

A good model will seek some sort of optimal relationship among the metrics. In this study, we seek to identify bankrupt organizations (recall) with some success without overclassifying non-bankrupt organizations. Assume that there are 100 organizations with 2 of them having declared bankruptcy. One estimator would be to assume that all organizations declared bankruptcy in the test set. That estimator would yield 100% recall but 2% accuracy. Another estimator might assume no organizations were bankrupt. While the estimator would be 98% accurate, it would have 0% recall. Thus, a balance between metrics is necessary.

## Results

### Descriptive analysis

Table 5 provides the descriptive statistics for fixed variables and for random variables offset at *y-1*. Variable descriptives at *y-2* and change between *y-2* and *y-1* are available online at https://rpubs.com/R-Minator/bf810. Also included are a large subset of the proposed prediction variables based on previously described research. NOTE: accounts receivable divided by net patient revenue was scaled by 1,000 and adjusted patient days was scaled by 10,000.

**Table 5:** Study variables

*n *= 3091	Mean	SD	Median	Skew	IQR	Q 0.25	Q 0.75
Bankrupt?	0.02	0.14	0.00	6.67	0.00	0.00	0.00
Year	2015.27	3.98	2016.00	−0.17	7.00	2012.00	2019.00
Government?	0.14	0.35	0.00	2.05	0.00	0.00	0.00
Urban?	0.10	0.30	0.00	2.62	0.00	0.00	0.00
For Profit?	0.24	0.43	0.00	1.24	0.00	0.00	0.00
Quality Stars = 1?	0.06	0.23	0.00	3.82	0.00	0.00	0.00
Quality Stars = 2?	0.20	0.40	0.00	1.53	0.00	0.00	0.00
Quality Stars = 4?	0.25	0.43	0.00	1.18	0.00	0.00	0.00
Quality Stars = 5?	0.12	0.33	0.00	2.30	0.00	0.00	0.00
Quality Stars = 4 or 5?	0.37	0.48	0.00	0.55	1.00	0.00	1.00
AR/Net Px Revenue (y-1)	3.75	2.53	4.14	0.04	4.31	1.49	5.80
% Medicaid Days (y-1)	0.09	0.09	0.06	2.85	0.08	0.03	0.11
% Medicare Days (y-1)	0.31	0.11	0.30	0.53	0.13	0.24	0.37
Current Ratio (y-1)	3.14	34.43	1.88	23.82	1.92	1.10	3.02
Debt: Equity (y-1)	2.94	101.39	0.32	35.01	0.79	0.02	0.81
Days Sales Outstanding (y-1)	55.68	113.35	48.26	16.28	16.20	40.83	57.03
Labor Compensation Ratio (y-1)	0.44	0.15	0.42	1.69	0.16	0.35	0.51
Operating Profit Margin (y-1)	−0.02	0.26	0.00	−7.54	0.15	−0.08	0.07
Days Cash On Hand (y-1)	47.73	128.65	13.42	7.22	53.60	0.16	53.76
Bed Utilization (y-1)	0.51	0.19	0.52	−0.20	0.29	0.36	0.65
Adjusted Patient Days (y-1)	8.92	9.80	5.98	3.39	8.87	2.77	11.64
Altman X1/Ohlson O3 (y-1)	−18304420.00	433505200.00	0.14	−26.67	0.23	0.04	0.27
Altman X2 (y-1)	−6227984.00	256046800.00	0.03	−52.81	0.12	−0.04	0.08
Altman X3 (y-1)	−945875.10	36316570.00	0.05	−27.92	0.14	−0.01	0.13
Altman X4 (y-1)	240771300.00	1653409000.00	0.06	4.88	0.31	−0.07	0.24
Ohlson O1.(y-1)	12.04	1.92	12.16	−5.70	1.84	11.23	13.07
Ohlson O2.(y-1)	3953532.00	101672000.00	0.45	31.67	0.47	0.24	0.71
Ohlson O4.(y-1)	0.56	4.71	0.49	−19.58	0.50	0.29	0.79
Ohlson O6.(y-1)	−1301918.00	40229010.00	0.04	−34.80	0.12	−0.01	0.11
Ohlson O7.(y-1)	302958500.00	1706703000.00	0.10	5.91	0.43	−0.03	0.40
Ohlson O9.(y-1)	8619685.00	131381800.00	2774170.00	20.28	18863185.50	−2832161.50	16031024.00
Ohlson O5.(y-1)	0.18	0.38	0.00	1.70	0.00	0.00	0.00
Ohlson O8.(y-1)	0.27	0.44	0.00	1.05	1.00	0.00	1.00

*Quality Stars =* 3 is the referent category and omitted

### Models

We considered many separate models to predict bankruptcy in hospitals. Table 6 provides a synopsis of those models.

**Table 6:** Summary of models

Model	Description
Model Group 1	Altman Models
Model 1a	Altman's Z'' with original coefficients at *y-1*
Model 1b	Altman's Z'' with re-estimated coefficients at *y-1*
Model Group 2	Ohlson Models
Model 2a	Ohlson's O with original coefficients at *y-1*
Model 2b	Ohlson's O with re-estimated coefficients at *y-1*
Model Group 3	Zmijewski Models
Model 3a	Zmijewski with original coefficients at *y-1*
Model 3b	Zmijewski with re-estimated coefficients at *y-1*
Model Group 4	BRKFSST Model

Our model of interest, the BRKFSST model, was titled based on the last names of the research group authors. Using the 12 most important standardized variables based on the magnitude of the absolute value of their coefficients and selected based on 3-Fold cross-validation of an ElasticNet GLM, we first estimated a logistic regression BRKFSST model. Only one Cook’s distance outlier was omitted from any of the training splits (the 50% split). Appendix 1 Table 15 is the metrics and coefficient comparison.

The mean balanced accuracy was 72.3% (SD = 6.3%), and the F1 Score averaged 0.135 across the splits. While low due to the extremely rare occurrences of bankruptcy, that average is far better than the next closest model with a F1-score of 0.086. The maximum misclassification rate is under 20% with a mean of 17.2% (SD = 2.2%) across all splits. Recall averaged 61.3% (SD = 12.6%) across the splits. AUC averages 83.9% (SD = 3.7%), which is the best of the models presented so far. The coefficients are directionally stable across all splits and of similar magnitude, although the change in Ohlson O2 and Altman X2 are both near zero (small effect size). The BRKFSST model estimated by logistic regression is the best performing model in class and by split. Appendix 1 Table 16 provides the log-odds estimates and associated statistical significance across splits.

Appendix 1 Table 16 illustrates that regardless of split, the coefficient estimates are stable and in the proper direction. When patient days at *y-2* increase, the probability of bankruptcy decreases. When accounts receivable divided by net patient revenue increases, the probability of bankruptcy decreases. Also, when labor compensation ratio increases, the probability of bankruptcy increases. A hospital that is owned by the government (public hospital) has a decreased probability of bankruptcy compared with privately owned hospitals. The variable directions all make sense.

Appendix 2 contains discussion and results from additional models we estimated, as well as results of the two excursions, COVID-19 and two-lagged variables. Results show dominance of the logistic regression approach, robustness to the COVID-19 period and need for further research on use of only 2-lagged explanatory variables.

### Overall model comparisons

With the number of models based on training split and variable selection, a review of each of the models’ performance is necessary.

Table 7 presents a thorough comparative evaluation of the predictive performance of multiple bankruptcy prediction models across various key metrics. The models evaluated include Altman’s original Z'', the re-estimated Altman Z'', Ohlson’s original O-score, the re-estimated Zmijewski model, and the newly developed BRKFSST model specifically tailored for the hospital industry.

**Table 7:** Evaluation of tested models

Metric	Altman Z''	Re-estimated Altman Z''	Ohlson's O	Re-estimated Zmijewski	BRKFSST
Balanced Accuracy	0.633	0.660	0.682	0.502	0.722
Precision	0.035	0.054	0.061	0.021	0.075
Recall	0.667	0.515	0.545	1.000	0.606
F1 Score	0.067	0.098	0.110	0.042	0.134
Specificity	0.599	0.804	0.818	0.003	0.838
Negative Predictive Value	0.988	0.987	0.988	1.000	0.990
Area Under the Curve	0.652	0.653	0.728	0.502	0.818
PR AUC	0.017	0.047	0.045	0.021	0.096
Misclassification Rate	0.399	0.202	0.188	0.975	0.167

The BRKFSST model clearly emerges as the most robust performer among those tested. With a balanced accuracy of 72.2%, it significantly exceeds the performance of traditional models, highlighting its improved ability to accurately identify both bankrupt and non-bankrupt hospitals. Its mean recall score of 60.6% demonstrates a notable capability to correctly identify actual bankruptcy cases. This attribute is crucial in financial risk prediction, where the cost of missing true positive cases (bankrupt hospitals incorrectly classified as solvent) is substantially high.

The model also achieves the highest Area Under the Curve (AUC) at 81.8%, further underscoring its superior discriminative ability compared to Altman’s Z'', re-estimated Altman Z'', Ohlson's O-score, and the Zmijewski model. Importantly, the BRKFSST model maintains a misclassification rate of only 16.7%, the lowest among all evaluated models. This indicates its balanced performance in minimizing both false positives and false negatives, crucial in the high-stakes healthcare context where accuracy has substantial real-world implications.

Conversely, the original Altman’s Z'' model, despite its historical relevance, consistently exhibited lower performance across multiple metrics. While it displayed reasonable recall (66.7%), this came at the expense of a very low precision (3.5%), indicating a substantial overclassification of bankruptcy events. The re-estimated Altman’s Z'' model showed improvement in specificity (80.4%) and reduced misclassification (20.2%), but still lagged significantly behind BRKFSST in balanced accuracy (66.0%) and AUC (65.3%).

Similarly, the original Ohlson’s O-score model (not shown) struggled, identifying no bankruptcies, highlighting its inadequacy when directly applied to hospital settings without recalibration. However, upon re-estimation, Ohlson’s model showed improved but still moderate performance (balanced accuracy of 68.2% and recall of 54.5%), suggesting that with proper calibration, its application could have merit but still substantially less than the BRKFSST model.

The re-estimated Zmijewski model demonstrated the poorest predictive capability overall, exhibiting a near-total misclassification rate (97.5%), negligible specificity (0.3%), and a recall of 100% but at the cost of precision (2.1%), rendering it ineffective in this context.

Overall, Table 7 emphasizes that traditional models, though historically significant, have limited predictive capacity when directly applied to the complex, unique financial environment of hospitals. The BRKFSST model not only outperforms these historical models but also provides interpretable insights critical for healthcare executives and policymakers, thereby offering a powerful tool for proactive financial management and strategic planning.

### Estimating the probability of bankruptcy for short term acute care hospitals

In balance, the BRKFSST model estimated by logistic regression performs the best among the models tested for predicting hospital bankruptcies based on F1-score, misclassification rates, and other metrics. Simplifying BRKFSST by removing zero coefficients and averaging across splits results in Eq. (6).

6 $$\begin{aligned}Log\;Odds\left(Bankruptcy\right)&=3.224-0.0878\times{Patient\;Days}_{y-2}\\&-0.360\left(\frac{{Acounts\;Receivable}_{y-1}}{{Net\;Patient\;Revenue}_{y-1}}\right)\\&-0.2146\left(\frac{{Acounts\;Receivable}_{y-2}}{{Net\;Patient\;Revenue}_{y-2}}\right)\\&-4.164\times{Bed\;Utilization}_{y-2}-0.0622\\&\times\Delta Liablities/Fund\;Balance\;Ratio+0.221\\&\times\Delta Ohlson\;O4-0.006\times{Days\;Cash}_{y-2}\\&-3.7248\times Star\;Rating{4or5}_{y-1}+2.4304\\&\times LaborCompensation{Ratio}_{y-1}-4.1500\\&\times\;Government\;Facility\end{aligned}$$

The above log odds are readily converted to probability through the function, *Pr(Bankruptcy)* = *exp(Log Odds)/(1* + *exp(Log Odds)*. This equation applied to the original bankruptcy formulation identifies 36 of the 65 bankrupt organizations correctly with a misclassification rate of 19% overall using a 0.5 or greater probability equating to a bankrupt status. Using a probability cut point of 0.44 or higher results in 44 of the 65 organizations being classified correctly, and a misclassification rate of 22%. Without converting to probabilities, the above equation can be used to estimate the likelihood of bankruptcy. Table 8 illustrates reasonable cut points for the log odds estimates.

**Table 8:** Score ranges for estimating bankruptcy likelihood

Values	Risk	# Bankruptcies
0.5 to Infinity	High	26
−0.5 to 0.49	Elevated	20
−3.00 to −0.49	Low	19
Below −3.00	Negligible	0

### Major findings

These results highlight three critical insights. First, the BRKFSST model substantially outperforms traditional financial models, achieving an AUC of 81.8% and balanced accuracy of 72.2%, while legacy approaches like Altman’s Z'', Ohlson’s O-score, and Zmijewski’s model failed to reliably identify bankrupt hospitals—sometimes missing all bankruptcy cases entirely. Second, the inclusion of hospital-specific variables such as HCAHPS quality ratings, adjusted patient days, and payer mix significantly enhanced predictive performance, confirming the value of tailoring models to the unique financial and operational realities of healthcare institutions. Finally, by employing a regularized logistic regression approach, the BRKFSST model balances predictive accuracy with interpretability, enabling hospital leaders and policymakers to not only identify at-risk organizations but also understand the drivers of financial vulnerability.

### Robustness test

As a test of the robustness of the BRKFSST Model, we matched 11 facilities in our dataset with Becker’s Hospital reports of bankruptcy for the year 2022 [35]. Using data from 2019 through 2020 only, we predicted the bankruptcy status of each facility based on the BRKFSST coefficients to build a confusion matrix. Since we previously documented the performance of BRKFSST for a one-year forward forecast, this evaluation served as a stress test of model performance two years forward. At the 0.5 cut point, BRKFSST correctly forecast 9 of the 11 bankrupt facilities. The precision was only 1%, however, meaning that for every 100 positives, 1 would actually be true. That is to be expected with hospital bankruptcy prediction (or any industry bankruptcy to be fair). Had we balanced the number of observations of bankrupt versus non-bankrupt facilities for this analysis as Ohlson did, our precision rate would have been about 82%.

## Discussion

### Relevance

In our study, we observed that traditional models such as Altman's Z'' and Ohlson’s O-score underperformed in the hospital setting, particularly when used without re-estimation or time-lagged variables. While Ohlson’s model showed some improvement with recalibration, it still lacked sufficient recall and interpretability in the healthcare context. In contrast, our BRKFSST logistic regression model demonstrated superior predictive performance, achieving an average Area Under the Curve (AUC) of 81.8%, balanced accuracy of 72.2%, and a mean recall of 60.6% across multiple test/train splits. These metrics suggest a strong capacity to discriminate between bankrupt and non-bankrupt hospitals, particularly in an imbalanced data context.

One of the most significant contributions of this model is its interpretability. While machine learning models such as neural networks and ensemble methods may outperform in raw accuracy, they offer little insight for hospital executives and policymakers who need actionable guidance. By deliberately employing logistic regression, our model reveals which factors most strongly influence the likelihood of bankruptcy and allows for individual hospital risk scores to be calculated. For example, the BRKFSST model identified several key predictors of bankruptcy: a higher labor compensation ratio was associated with increased risk, while stronger patient volumes (e.g., adjusted patient days) and government ownership reduced the likelihood of bankruptcy. Additionally, lower scores in quality metrics, such as the Hospital Consumer Assessment of Healthcare Providers and Systems (HCAHPS) Star Ratings, were also associated with financial distress.

### Managerial implications

It is crucial for all organizations, and more precisely hospitals, to monitor their financial and quality performance metrics and detect early signs of financial trouble to prevent bankruptcy. Our findings present clear and actionable implications for hospital leaders. Organizations that operate with tighter margins, particularly those with high labor costs and low-quality ratings, should be closely monitored. Leadership teams can use the derived equation to generate risk scores based on their own institutional data, enabling proactive financial stewardship and contingency planning. Moreover, policy makers may find the model useful in identifying facilities that require financial support or regulatory interventions, especially those in underserved or economically vulnerable areas. Overall, this research advances the field by combining predictive accuracy with interpretability in a high-stakes environment. While no model can perfectly forecast future insolvency, this study offers a valuable tool for mitigating risk in a sector where bankruptcy has direct implications for public health.

Through the development of a hospital-specific model that encompasses reliable predictive factors of bankruptcy, we seek to provide health policy makers and leaders of healthcare institutions with the insight necessary to take proactive steps to avoid financial distress and bankruptcy filing as well as ensure long-term financial viability at the organizational level. We suggest our model can be used to assess individual hospital financial risk based on the parameter estimates we have developed, and we propose a relatively straight-forward calculation to indicate distress. Ultimately, our plan is to evaluate all short-term acute care hospitals in the US using the BRKFSST model to discern which hospitals are most at risk.

### Limitations and suggestions for future research

Our study is limited in several ways. First, our study provides indication of the most relevant factors that are associated with hospital bankruptcy, but our modeling falls short of providing hospital leaders with the exact values beyond which organizational solvency is impossible to sustain. Future work could focus on this shortcoming to provide hospital leaders with granular insight into the ‘critical few’ variables we have found in the current study and to provide insight into the ‘tipping point’ for each [36]. Further, it would also be useful to provide a nationwide analysis of hospitals, given the parameters identified in this study, to identify which hospitals are most at risk of failure. This might provide legislators and policymakers with a useful tool to direct preemptive action and ensure access to care is assured for patients and families served by at risk hospitals.

There may be other factors with a significant influence on bankruptcy that we did not consider in our study. This is apparent in our lower-than-desired precision and recall values in each of our final analytical models. Additional variables we could consider in the future might include hospitals’ bond ratings, more detailed study of hospital payer mix, and extended study of local market conditions around each facility. Notably, we acknowledge that our current study does not include specifics pertaining to the demographics or socioeconomics of each hospital’s local patient population. Although we have included some associated proxies for these factors in our study (i.e., urban/rural, Medicaid days, DSH payments, etc.), none of these are as accurate as we would like them to be given their likely connection to the financial health of the hospital. We conjecture that hospitals located in areas facing challenging economic conditions are more prone to be financially troubled. Hospitals in depressed areas might experience lower levels of philanthropic and charitable support in addition to less robust commercial insurance coverage. Without question, examining this set of variables would require a more detailed and comprehensive dataset of facility and market-level data, but we believe it would add substantive depth and quality to the study.

Another limitation of our study is that bankruptcy filing is inherently a dichotomous variable. Thus, our study does not capture those hospitals that are near bankruptcy or in other stages of financial distress other than via probabilistic ranges. We conjecture that there may be some hospitals in our study that faced financial difficulty and were near bankruptcy but found other sources of capital or altered operations sufficiently to avoid the bankruptcy process. This may be a contributory factor to the misclassification of bankrupt hospitals we found. Future work may benefit from considering something other than a dichotomous outcome or evaluating stages of organizational financial distress as the dependent variable. Furthermore, other factors such as politics may play an important role in bankruptcy filing, especially for government/owned hospitals. Our finding indicated that government-owned hospitals have a lower probability of filing for bankruptcy compared with their privately-owned counterparts, though they usually face more financial problems [3].

Also, this study relies on publicly reported data from several thousand facilities compiled over a fifteen-year period. Although the length of time we have studied is a strength of our study, we are not able to verify the accuracy of the data beyond what is reported to the American Hospital Association, CMS, and other agencies. Future work could be based on a primary data collection effort that captures not just the data elements that are publicly reported but could verify the accuracy and authenticity of the data from each host site. This deeper investigation could feature issues of local, state, and federal policy, local market macroeconomic trends, regional inflationary pressures, and other related factors. A collected sample of data from hospitals at this level of granularity would assist us in more accurately capturing the subtle nuances of the factors that undermine or strengthen hospital financial health. Without question, this would be a much more difficult endeavor than what we have developed, but it is worth considering given the impact that hospital bankruptcies, and subsequent closures, have on communities across the country.

This is a study strictly focused on hospitals in the United States. Future research might consider extending the study to other countries to facilitate cross-country comparisons. We should note that the business and operational environment of US hospitals is far different than any other industry and certainly different than healthcare in almost any other developed nation—in terms of how hospitals generate revenue, their varied ownership structures, taxation implications, and regulatory requirements. In our opinion, there is not a more convoluted or complex operating environment on the planet than the US hospital industry. Thus, making international comparisons might prove difficult, but it is worth considering given the potential federal and state level policy guidance that might result from such an examination.

It has been said, “prediction is very hard, especially if it’s about the future.” While the study proffers interpretable models that outperform some traditional models for predicting hospital bankruptcy on unseen tests sets, the model is by no means perfect. No guarantee exists that this model will be robust enough to handle the future environment. The BRKFSST model deliberately employed interpretable estimation of a fixed model. Other models we ran included random forests, gradient boosted ensembles, neural networks, etc. Many of these types of models outperformed BRKFSST but provided no actionable information for decision makers and little interpretability. Since the goal of this research was to provide an interpretable model, we restricted our analysis to logistic regression. In forthcoming research, we will provide less interpretable but more precise models with better recall that would potentially inform funding decision makers rather than board members, investors, the public, and others interested in explainable models.

## Conclusions

This study developed and validated a hospital-specific bankruptcy prediction model, BRKFSST, which significantly outperforms widely-used legacy financial models like Altman’s Z'', Ohlson’s O-score, and Zmijewski’s model. By incorporating both traditional financial indicators and sector-specific variables—such as quality ratings, payer mix, and hospital utilization measures—BRKFSST achieves greater predictive accuracy and interpretability, making it a powerful tool for anticipating financial distress in U.S. hospitals.

These findings underscore the importance of developing domain-aware models in high-stakes sectors like healthcare. Unlike generic financial tools, BRKFSST provides insights that are not only statistically robust but also actionable, helping hospital leaders, boards, and policymakers make timely decisions to prevent insolvency. This is especially vital in rural and safety-net facilities, where unexpected closures can have catastrophic consequences for community health outcomes and service accessibility.

Looking ahead, future research should validate BRKFSST on post-pandemic datasets, explore integration with machine learning methods for enhanced risk stratification, and assess implementation feasibility across diverse hospital systems. We also encourage replication and extension of this approach in other vulnerable sectors—such as nursing homes, rural clinics, and behavioral health providers—where financial instability threatens essential services. Open, interpretable, and data-informed tools like BRKFSST are critical for safeguarding institutional resilience and ensuring continued patient access in an evolving healthcare landscape.

## Figures and Tables

**Fig. 2:**
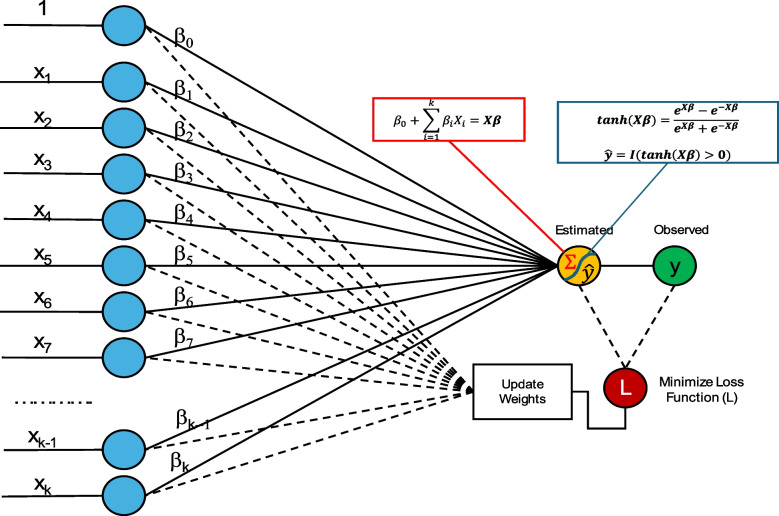
Example perceptron

**Fig. 3:**
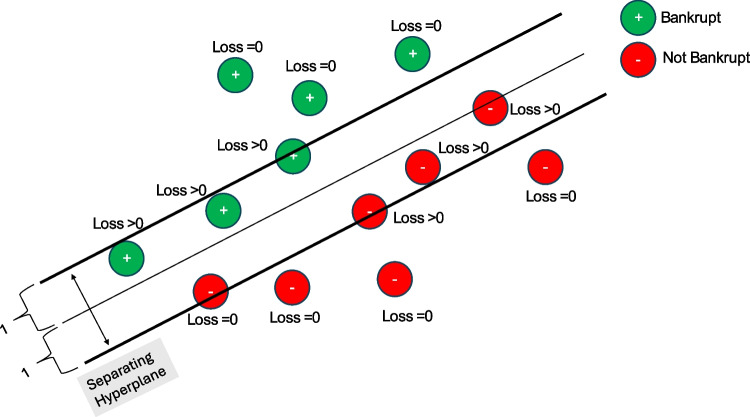
Separating hyperplane example (derived from Beauvais et. al, 2022)

## Data Availability

The datasets analyzed in this study are derived from publicly available sources and/or proprietary databases as described in the Methods section. The statistical code used to derive the results presented in this article are available at https://rpubs.com/R-Minator/bf810. Specifically, the bankruptcy and financial indicator data were obtained from Definitive Healthcare, which can be accessed at https://www.definitivehc.com/ . Due to data use agreements and confidentiality provisions associated with certain proprietary hospital financial datasets, access to raw individual-level records is restricted; researchers may request access by contacting the corresponding author or through the original data provider, subject to approval and compliance with applicable data use terms.

## Appendix 1 Detailed results of models tested

This appendix provides detailed performance diagnostics for models evaluated in this study, including traditional financial models and the newly developed BRKFSST model. These details are included here to promote transparency, reproducibility, and comparability.

**Table 9:** Altman Z’’ accuracy metrics by split (PR-AUC = Precision/Recall Area Under the Curve, SD = Standard Deviation)

Metric	50%	60%	70%	80%	90%	Mean	SD
Balanced Accuracy	0.633	0.614	0.567	0.632	0.714	0.632	0.047
Precision	0.035	0.032	0.028	0.033	0.044	0.034	0.005
Recall	0.667	0.654	0.550	0.692	0.857	0.684	0.099
F1 Score	0.067	0.061	0.054	0.064	0.084	0.066	0.010
Specificity	0.599	0.574	0.585	0.571	0.571	0.580	0.011
Negative Predictive Value	0.988	0.987	0.983	0.989	0.994	0.988	0.004
Area Under the Curve	0.652	0.634	0.582	0.623	0.785	0.655	0.069
PR AUC	0.017	0.021	0.024	0.020	0.039	0.024	0.008
Misclassification Rate	0.399	0.425	0.416	0.426	0.423	0.418	0.010

**Table 10:** Re-estimated Altman’s Z'' accuracy metrics and coefficient estimates (Log Odds) by Split (PR-AUC = Precision/Recall Area Under the Curve, SD = Standard Deviation)

Metric/coefficient	50%	60%	70%	80%	90%	Mean	SD
Balanced Accuracy	0.660	0.672	0.462	0.536	0.465	0.559	0.091
Precision	0.054	0.051	0.020	0.023	0.021	0.034	0.015
Recall	0.515	0.577	0.850	1.000	0.857	0.760	0.184
F1 Score	0.098	0.093	0.039	0.044	0.041	0.063	0.027
Specificity	0.804	0.767	0.074	0.073	0.073	0.358	0.349
Negative Predictive Value	0.987	0.988	0.957	1.000	0.957	0.978	0.018
Area Under the Curve	0.653	0.632	0.485	0.535	0.527	0.566	0.065
PR AUC	0.047	0.042	0.073	0.022	0.172	0.071	0.053
Misclassification Rate	0.202	0.237	0.909	0.908	0.910	0.633	0.338
(Intercept)	-0.216	0.048	0.026	0.031	0.031	-0.016	0.100
Altman X1 (y-1)	0.013	-1.169	0.000	0.000	0.000	-0.231	0.469
Altman X2 (y-1)	0.096	0.061	0.000	0.000	0.000	0.031	0.040
Altman X3 (y-1)	-6.005	-1.282	0.000	0.000	0.000	-1.457	2.327
Altman X4 (y-1)	0.000	0.000	0.000	0.000	0.000	0.000	0.000

**Table 11:** Model coefficient comparison table

Variable	50%	60%	70%	80%	90%
(Intercept)	-0.216 (0.043)***	0.048 (0.036)	0.026 (0.031)	0.031 (0.029)	0.031 (0.028)
Altman X1 (y-1)	0.013 (0.101)***	-1.169 (0.114)***	0.000 (< 0.001)	0.000 (< 0.001)	0.000 (< 0.001)
Altman X2 (y-1)	0.096 (0.089)	0.061 (0.105)	0.000 (< 0.001)	0.000 (< 0.001)	0.000 (< 0.001)
Altman X3 (y-1)	-6.005 (0.315)***	-1.282 (0.17)***	0.000 (< 0.001)	0.000 (< 0.001)	0.000 (< 0.001)
Altman X4 (y-1)	0.000 (< 0.001)**	0.000 (< 0.001)*	0.000 (< 0.001)***	0.000 (< 0.001)***	0.000 (< 0.001)***

Log Odds (Standard Error), + = *p* < 0.10, * = *p* < 0.05, ** = *p* < 0.01,*** = *p* < 0.001

**Table 12:** Ohlson’s O accuracy metrics and coefficient estimates (Log Odds) by Split (PR-AUC = Precision/Recall Area Under the Curve, SD = Standard Deviation)

Metric/coefficient	50%	60%	70%	80%	90%	Mean	SD
Balanced Accuracy	0.682	0.587	0.607	0.695	0.660	0.646	0.042
Precision	0.061	0.035	0.040	0.047	0.050	0.047	0.009
Recall	0.545	0.423	0.450	0.692	0.571	0.536	0.096
F1 Score	0.110	0.065	0.074	0.088	0.092	0.086	0.015
Specificity	0.818	0.751	0.764	0.698	0.749	0.756	0.038
Negative Predictive Value	0.988	0.984	0.984	0.991	0.987	0.987	0.003
Area Under the Curve	0.728	0.712	0.704	0.749	0.765	0.732	0.023
PR AUC	0.045	0.035	0.038	0.045	0.185	0.070	0.058
Misclassification Rate	0.188	0.256	0.242	0.302	0.255	0.249	0.037
(Intercept)	19.096	30.803	28.249	24.405	29.772	26.465	4.277
Ohlson O1. (y-1)	-8.015	-12.768	-11.834	-9.973	-12.383	-10.995	1.772
Ohlson O2. (y-1)	0.378	0.000	0.000	0.000	0.000	0.076	0.151
Ohlson O3 (y-1)	0.199	0.000	0.000	0.000	0.000	0.040	0.080
Ohlson O4. (y-1)	0.056	0.211	0.135	0.172	0.094	0.134	0.055
Ohlson O5. (y-1)	-1.907	-0.173	-1.203	-1.371	-0.596	-1.050	0.606
Ohlson O6. (y-1)	-5.228	0.000	0.000	0.000	0.000	-1.046	2.091
Ohlson O7. (y-1)	0.000	0.000	0.000	0.000	0.000	0.000	0.000
Ohlson O8. (y-1)	0.587	0.096	1.209	-0.037	0.616	0.494	0.442
Ohlson O9. (y-1)	0.000	0.000	0.000	0.000	0.000	0.000	0.000

**Table 13:** Ohlson’s O model coefficient comparison table

Variable	50%	60%	70%	80%	90%
(Intercept)	19.096 (1.338)***	30.803 (1.197)***	28.249 (1.115)***	24.405 (0.931)***	29.772 (0.946)***
Ohlson O1 (y-1)	-8.015 (0.543)***	-12.768 (0.49)***	-11.834 (0.458)***	-9.973 (0.379)***	-12.383 (0.389)***
Ohlson O2 (y-1)	0.378 (0.111)**	0.000 (< 0.001)	0.000 (< 0.001)	0.000 (< 0.001)	0.000 (< 0.001)
Ohlson O3 (y-1)	0.199 (0.103) +	0.000 (< 0.001)	0.000 (< 0.001)	0.000 (< 0.001)	0.000 (< 0.001)
Ohlson O4 (y-1)	0.056 (0.029) +	0.211 (0.038)***	0.135 (0.027)***	0.172 (0.025)***	0.094 (0.02)***
Ohlson O5 (y-1)	-1.907 (0.162)***	-0.173 (0.108)	-1.203 (0.122)***	-1.371 (0.112)***	-0.596 (0.093)***
Ohlson O6 (y-1)	-5.228 (0.551)***	0.000 (< 0.001)	0.000 (< 0.001)	0.000 (< 0.001)	0.000 (< 0.001)
Ohlson O7 (y-1)	0.000 (< 0.001)	0.000 (< 0.001)	0.000 (< 0.001)	0.000 (< 0.001)	0.000 (< 0.001)
Ohlson O8 (y-1)	0.587 (0.114)***	0.096 (0.088)	1.209 (0.079)***	-0.037 (0.075)	0.616 (0.071)***
Ohlson O9 (y-1)	0.000 (< 0.001)	0.000 (< 0.001)	0.000 (< 0.001)**	0.000 (< 0.001)***	0.000 (< 0.001)***

+ = *p* < 0.10, * = *p* < 0.05, ** = *p* < 0.01, *** = *p* < 0.001

**Table 14:** Zmijewski accuracy metrics and coefficient estimates (Log Odds) by Split (PR-AUC = Precision/Recall Area Under the Curve, SD = Standard Deviation)

Metric/coefficient	50%	60%	70%	80%	90%	Mean	SD
Balanced Accuracy	0.502	0.571	0.586	0.566	0.655	0.576	0.055
Precision	0.021	0.028	0.030	0.026	0.035	0.028	0.005
Recall	1.000	0.538	0.600	0.692	0.857	0.737	0.190
F1 Score	0.042	0.054	0.057	0.050	0.067	0.054	0.009
Specificity	0.003	0.603	0.572	0.439	0.452	0.414	0.241
Negative Predictive Value	1.000	0.984	0.985	0.985	0.993	0.989	0.007
Area Under the Curve	0.502	0.623	0.653	0.616	0.779	0.635	0.099
PR AUC	0.021	0.088	0.091	0.050	0.141	0.078	0.045
Misclassification Rate	0.975	0.399	0.428	0.556	0.539	0.579	0.231
(Intercept)	0.004	0.367	0.434	0.033	0.054	0.178	0.205
Z1 = Ohlson O6 (y-1)	0.000	0.000	0.000	0.000	0.000	0.000	0.000
Z2 = Ohlson O2 (y-1)	0.000	0.000	0.000	0.000	0.000	0.000	0.000
Z3 = 1/Ohlson O4 (y-1)	0.000	-0.274	-0.311	-0.020	-0.035	-0.128	0.151

**Table 15:** BRKFSST accuracy metrics and coefficient estimates (Log Odds)(PR-AUC = Precision/Recall Area Under the Curve, SD = Standard Deviation)

Metric/coefficient	50%	60%	70%	80%	90%	Mean	SD
Balanced Accuracy	0.722	0.737	0.620	0.748	0.789	0.723	0.063
Precision	0.075	0.072	0.052	0.070	0.109	0.076	0.021
Recall	0.606	0.654	0.400	0.692	0.714	0.613	0.126
F1 Score	0.134	0.130	0.092	0.128	0.189	0.135	0.035
Specificity	0.838	0.819	0.840	0.804	0.865	0.833	0.023
Negative Predictive Value	0.990	0.991	0.985	0.992	0.992	0.990	0.003
Area Under the Curve	0.818	0.833	0.793	0.860	0.889	0.839	0.037
PR AUC	0.096	0.073	0.066	0.200	0.094	0.106	0.054
Misclassification Rate	0.167	0.184	0.169	0.199	0.139	0.172	0.022
(Intercept)	2.388	4.195	3.068	3.175	3.306	3.226	0.647
Patient Days (y-2)	-0.065	-0.072	-0.116	-0.076	-0.110	-0.088	0.023
Altman X2 (y-2)	0.000	0.000	0.000	0.000	0.000	0.000	0.000
AR/Op Income (y-1)	-0.346	-0.283	-0.436	-0.373	-0.362	-0.360	0.055
AR/Op Income (y-2)	-0.141	-0.366	-0.257	-0.095	-0.214	-0.215	0.106
Bed Utilization (y-2)	-4.867	-5.302	-3.058	-3.774	-3.819	-4.164	0.906
Change in Liability to Fund Balance	-0.014	-0.162	-0.115	-0.006	-0.014	-0.062	0.072
Change in Ohlson O4	0.505	0.097	0.181	0.150	0.172	0.221	0.162
Days Cash-on-Hand (y-2)	-0.007	-0.011	-0.003	-0.005	-0.004	-0.006	0.003
4 or 5 Star Facility (y-1)	-4.862	-2.980	-3.681	-3.620	-3.481	-3.725	0.693
Labor Compensation Ratio (y-1)	4.106	1.764	2.516	1.806	1.960	2.431	0.984
Government?	-5.538	-3.123	-4.461	-3.372	-4.256	-4.150	0.961
Change in Ohlson O2	0.000	0.000	0.000	0.000	0.000	0.000	0.000

**Table 16:** BRKFSST Model Coefficient Comparison Table

Variable	50%	60%	70%	80%	90%	Votes
(Intercept)	2.388 (0.333)***	4.195 (0.342)***	3.068 (0.294)***	3.175 (0.253)***	3.306 (0.245)***	2.4 and 4.2
Patient Days (y-2)	-0.065 (0.017)***	-0.072 (0.016)***	-0.116 (0.015)***	-0.076 (0.011)***	-0.11 (0.013)***	All Negative
Altman X2 (y-2)	0.000(< 0.001)	0.000(< 0.001)	0.000(< 0.001)***	0.000(< 0.001)	0.000(< 0.001)	All Near Zero
AR/Op Income(y-1)	-0.346 (0.035)***	-0.283 (0.03)***	-0.436 (0.03)***	-0.373 (0.026)***	-0.362 (0.024)***	All Negative
AR/Op Income(y-2\)	-0.141 (0.036)***	-0.366 (0.031)***	-0.257 (0.029)***	-0.095 (0.025)***	-0.214 (0.025)***	All Negative
Bed Utilization (y-2)	-4.867 (0.435)***	-5.302 (0.399)***	-3.058 (0.354)***	-3.774 (0.287)***	-3.819 (0.304)***	All Negative
Change in Liability: Fund Balance	-0.014 (0.013)	-0.162 (0.03)***	-0.115 (0.03)***	-0.006 (0.001)***	-0.014 (0.005)**	All Negative
Change in Ohlson O4	0.505 (0.056)***	0.097 (0.016)***	0.181 (0.033)***	0.15 (0.023)***	0.172 (0.023)***	All Positive
Days Cash-on-Hand (y-2)	-0.007 (0.001)***	-0.011 (0.001)***	-0.003 (0.001)*	-0.005 (0.001)***	-0.004 (0.001)***	All Negative
4 or 5 Star Rating (y-1)	-4.862 (0.384)***	-2.98 (0.181)***	-3.681 (0.209)***	-3.62 (0.189)***	-3.481 (0.181)***	All Negative
Labor Compensation Ratio (y-1)	4.106 (0.567)***	1.764 (0.494)***	2.516 (0.44)***	1.806 (0.381)***	1.960 (0.390)***	All Positive
Government Status	-5.538 (0.436)***	-3.123 (0.215)***	-4.461 (0.288)***	-3.372 (0.201)***	-4.256 (0.247)***	All Negative
Change in Ohlson O2	0.000(< 0.001)	0.000(< 0.001)**	0.000(< 0.001)	0.000(< 0.001)	0.000(< 0.001)***	AllNear Zero

+ = *p* < 0.10, * = *p* < 0.05, ** = *p* < 0.01, *** = *p* < 0.001

## Appendix 2 Additional models and excursions examined

Aside from the models reviewed throughout the body of this paper, the research team investigated several additional models and two excursions. Additional models included a Perceptron, Linear Support Vector Machine***,*** and a Unique Variable Decision Tree. The first excursion applied to the COVID period (year 2019 through 2021) only. The intent was to investigate the robustness of the estimated model for that given period given potential changes in the environment. The second excursion involved building a model that used two-lagged variables only. The intent of this model was to evaluate its performance given that early identification of potential issues allows for intervention quicker.

### Perceptron for BRKFSST model

We used a modified Perceptron for alternatively estimating the BRKFSST model. A Perceptron is a simple neural network with a single layer with one node for each input variable plus a bias input (Appendix 2 Fig. 2). Weights connect these nodes with the output node. Those weights are multiplied by the observations and summed, and then an activation function introduces non-linearity. In Appendix 2 Fig. 2, a hyperbolic tangent function (blue symbol) is shown with the aggregate weights (red summation symbol). A loss function is then minimized, and the weights are tuned through optimization. While the Perceptron model does require scaling all variables (e.g., min–max scaling between 0 and 1 or standard scaling), the method provides weights of coefficients (e.g., the $\beta$ coefficients in the diagram) which are readily interpreted for both directionality and magnitude.

In the case of the BRKFSST model, we used the hyperbolic tangent as our activation function and the sum of the squared error as our loss function. Often a logistic function is used with binary cross-entropy for 2-class classification. However, using a logistic function might generate the same results as a logistic regression. As shown in Equation (Appendix 1 Eq. 7), where *y* is the vector of true observations, *p* is the vector of predicted probability of bankruptcy, *n* is the number in the sample, and $\beta$ is the vector of parameter estimates, the BCE is simply the negative of the log-likelihood. Minimizing BCE maximizes the log-likelihood. The use of a different activation function, loss function, and optimization method might generate different and potentially more interesting results.

7 $$BCE=-\frac{1}{n}\left[y\bullet \mathrm{log}\left(p\right)+\left(1-y\right)\bullet \mathrm{log}\left(1-p\right)\right]=-\frac{\mathcal{L}(\beta )}{n}$$

The re-estimated BRKFSST model used weights generated from a Perceptron. To provide an alternative estimation to the logistic regression model, the Perceptron used a hyperbolic tangent error function with a sum of the squared errors loss function. Predictors were scaled, and all observations from the training set were included in a single batch. After three epochs, the model showed no material changes in performance for any of the training splits (Akaike Information Criterion). The *neuralnet* library in R provided the functions for the analysis. To generate a Perceptron, we only required the weights and bias of the single hidden node (necessary for the *neuralnet* library) to have a bias of zero and an output weight of 1.0. Each training/test split pair were evaluated. The confusion matrix suggested confusing and inconsistent results among splits. For the 50% split, all but two observations were classified as bankrupt, thus the model was unusual. The 60% split identified 5 out of 26 bankruptcies and 1172 out of 1211 non-bankruptcies, which was not very good. The 70% split correctly classified no bankruptcies, and the 80% split classified 460 out of the 619 observations as bankruptcies. The 90% split classified all but two observations as bankrupt. Looking at the estimation across models, the Perceptron model is highly sensitive to the split size, and not a useful model. Since the model results are dependent on the split, the Perceptron estimation was not considered further.

### Linear support vector machine for BRKFSST

A second method used for estimating the BRKFSST model was a linear support vector machine (SVM) with a hinge-loss function. Linear SVM seeks to find the maximum separating hyperplane between groups by fixing two parallel lines (planes) that separate each group and maximizing the difference between them. Assume that bankruptcy status is coded -1 for no bankruptcy and + 1 for bankruptcy. Then SVM looks for the minimization solution to the following Lagrangian problem (Appendix 2 Eq. 8), a solution which minimizes the weights (coefficients) for the parallel lines (planes) created by the separating hyperplane, which is the equivalent (primal/dual) of maximizing the separating hyperplane.

8 $$\mathcal{L}\left(w,b,\lambda \right)=\frac{1}{2}{w}^{T}w-{\lambda }^{t}[Y\left(Xw-b1\right)-\gamma \bullet 1]$$

In Appendix 2 Eq. 8, ***w*** are the weights associated with the predictors, ***Y*** is a diagonal matrix with the dependent variable (bankruptcy {-1,1} on the diagonal, ***X*** is the matrix of predictors, *b* is the intercept, ***1*** is a matrix of 1’s, ${\boldsymbol{\lambda}}$ are the Lagrangian multipliers, and $\gamma$ is the margin size (somtimes fixed). Minimizing this equation results in a maximal separation of the two parallel lines (planes) that are the hyperplane depicted in Appendix 2 Fig. 3.

The linear SVM produces coefficient estimates useful for assessing directionality and magnitude of effects. The variables must be standardized (e.g., min max transformation). However, this set of models proved unreliable. The SVM classified nearly all observations as bankrupt for the 50% split, a reasonable split for the 60% split, all not bankrupt for the 70% split, nearly all bankrupt for the 80% split, and all but 2 bankrupt for the 90% split. The model is sensitive to training/test splits, and thus it was not considered further.

### Unique variable decision tree for BRKFSST

An additional estimation model was a tree-based model designed to retain interpretability, a model we term Unique Variable Decision Tree (UVDT). This model builds a decision tree that allows each variable to enter only one time on each major branch (left or right) based solely on the Gini coefficient. We investigated other decision metrics as well. Indicator functions for splits are readily interpretable, and by not re-using variables on the two separate branches, there are no unexplainable nonlinearities. Discretization of variables may produce results unseeable when using parametric methods, particularly when the assumptions of the parametric models are not fully met.

This method for estimating the BRKFSST model leveraged a custom-design decision tree model (UVDT). This model allowed for variables to enter by Gini importance only one time on each major branch (left or right). Doing so created a set of indicator variables for easy interpretation. Such a tree limits the ability to capture nonlinearities, so the trade space between interpretation and performance leans towards the former. The tree model was built manually using R. Appendix 2 Table 17 shows the accuracy metrics.

**Table 17:** Accuracy metrics and coefficient estimates, UVDT

Metric/coefficient	50%	60%	70%	80%	90%	Mean	SD
Balanced Accuracy	0.638	0.621	0.596	0.687	0.657	0.640	0.035
Precision	0.059	0.055	0.047	0.052	0.079	0.058	0.012
Recall	0.424	0.385	0.350	0.615	0.429	0.441	0.103
F1 Score	0.104	0.096	0.082	0.096	0.133	0.102	0.019
Specificity	0.853	0.857	0.843	0.759	0.884	0.839	0.047
Negative Predictive Value	0.985	0.985	0.983	0.989	0.985	0.986	0.002
Area Under the Curve	0.591	0.642	0.598	0.703	0.739	0.654	0.065
PR AUC	0.040	0.039	0.033	0.050	0.059	0.044	0.010
Misclassification Rate	0.157	0.153	0.168	0.244	0.126	0.169	0.044

The UDVT confusion matrices appeared somewhat stable across the splits, balancing recall and specificity. The metrics in Appendix 2 Table 17 confirmed the stability. The balanced accuracy average across splits is 64%, and the average recall is 44.1%. The mean F1 Score across splits is lower than that of prior models at 0.102 (SD = 0.019); however, the misclassification rate is lower for each split with the exception of the 80% split.. Thus, this model appears to be useful..

### Model excursion 1, COVID

The BRKFSST model was applied solely to the bankruptcies that occurred during COVID as an excursion. The intent was to evaluate the robustness of the model for a specific time where factors influencing bankruptcy might have changed. Each of the models trained on the various splits were applied to data filtered for 2019 through 2021. The BRKFSST model appeared to be robust for that time period as indicated by Appendix 2 Table 18 (accuracy metrics).

**Table 18:** Accuracy Metrics for COVID Excursion

Metric/coefficient	50%	60%	70%	80%	90%	Mean	SD
Balanced Accuracy	0.688	0.752	0.693	0.671	0.620	0.685	0.048
Precision	0.023	0.027	0.025	0.020	0.018	0.023	0.004
Recall	0.571	0.714	0.571	0.571	0.429	0.571	0.101
F1 Score	0.045	0.052	0.047	0.038	0.035	0.043	0.007
Specificity	0.804	0.790	0.815	0.771	0.811	0.798	0.018
Negative Predictive Value	0.996	0.997	0.996	0.995	0.994	0.996	0.001
Area Under the Curve	0.757	0.716	0.696	0.761	0.722	0.730	0.028
PR AUC	0.018	0.021	0.017	0.018	0.016	0.018	0.002
Misclassification Rate	0.197	0.210	0.187	0.231	0.192	0.203	0.018

In Appendix 1 Table 10, the mean balanced accuracy is 68.5% (SD = 4.8%). The recall is reasonable (57.1% mean), and the precision is predictably low.

### Model excursion 2, two-year lagged variables only

As an additional excursion, we ran a GLM model that used two-year lagged data and fixed variables only. The intent was to see whether such a model might be able to provide additional and earlier insight into hospital bankruptcies. Appendix 2 Table 19 shows the accuracy metrics and coefficient estimates. The results of the excursion show promise for early identification of bankruptcy. In balance, a 2-lag + model appears to be feasible and will be the object of further investigation.

**Table 19:** Accuracy Metrics for 2-Lag Excursion

Metric/coefficient	50%	60%	70%	80%	90%	Mean	SD
Balanced Accuracy	0.654	0.656	0.566	0.682	0.488	0.609	0.081
Precision	0.057	0.039	0.045	0.039	0.021	0.040	0.013
Recall	0.485	0.654	0.250	0.769	0.429	0.517	0.201
F1 Score	0.101	0.074	0.076	0.075	0.041	0.073	0.021
Specificity	0.824	0.657	0.882	0.596	0.548	0.701	0.145
Negative Predictive Value	0.987	0.989	0.982	0.992	0.976	0.985	0.006
Area Under the Curve	0.654	0.656	0.566	0.682	0.488	0.609	0.081
PR AUC	0.043	0.035	0.031	0.037	0.022	0.034	0.008
Misclassification Rate	0.184	0.343	0.131	0.401	0.455	0.303	0.140

## Declarations

### Research involving humans

No human subjects were used in this research
